# In-depth transcriptomic analysis of *Anopheles gambiae* hemocytes uncovers novel genes and the oenocytoid developmental lineage

**DOI:** 10.1186/s12864-024-09986-6

**Published:** 2024-01-19

**Authors:** Banhisikha Saha, Colton M McNinch, Stephen Lu, Margaret C.W. Ho, Stephanie Serafim De Carvalho, Carolina Barillas-Mury

**Affiliations:** 1 Laboratory of Malaria and Vector Research, National Institute of Allergy and Infectious Diseases, National Institutes of Health, Rockville, USA; 2 Bioinformatics and Computational Biosciences Branch, Office of Cyber Infrastructure and Computational Biology, National Institute of Allergy and Infectious Diseases (NIAID), National Institutes of Health, 20892 Bethesda, MD USA; 3 Vector Biology Section, Laboratory of Malaria and Vector Research, National Institute of Allergy and Infectious Diseases, Bethesda, MD USA

**Keywords:** Hemocytes, Transcriptome, RNA-seq, Cellular immunity, Mosquito, Anopheles gambiae

## Abstract

**Background:**

Hemocytes are immune cells that patrol the mosquito hemocoel and mediate critical cellular defense responses against pathogens. However, despite their importance, a comprehensive transcriptome of these cells was lacking because they constitute a very small fraction of the total cells in the insect, limiting the study of hemocyte differentiation and immune function.

**Results:**

In this study, an in-depth hemocyte transcriptome was built by extensive bulk RNA sequencing and assembly of hemocyte RNAs from adult *A. gambiae* female mosquitoes, based on approximately 2.4 billion short Illumina and about 9.4 million long PacBio high-quality reads that mapped to the *A. gambiae* PEST genome (P4.14 version). A total of 34,939 transcripts were annotated including 4,020 transcripts from novel genes and 20,008 novel isoforms that result from extensive differential splicing of transcripts from previously annotated genes. Most hemocyte transcripts identified (89.8%) are protein-coding while 10.2% are non-coding RNAs. The number of transcripts identified in the novel hemocyte transcriptome is twice the number in the current annotation of the *A. gambiae* genome (P4.14 version). Furthermore, we were able to refine the analysis of a previously published single-cell transcriptome (scRNAseq) data set by using the novel hemocyte transcriptome as a reference to re-define the hemocyte clusters and determine the path of hemocyte differentiation. Unsupervised pseudo-temporal ordering using the Tools for Single Cell Analysis software uncovered a novel putative prohemocyte precursor cell type that gives rise to prohemocytes. Pseudo-temporal ordering with the Monocle 3 software, which analyses changes in gene expression during dynamic biological processes, determined that oenocytoids derive from prohemocytes, a cell population that also gives rise to the granulocyte lineage.

**Conclusion:**

A high number of mRNA splice variants are expressed in hemocytes, and they may account for the plasticity required to mount efficient responses to many different pathogens. This study highlights the importance of a comprehensive set of reference transcripts to perform robust single-cell transcriptomic data analysis of cells present in low abundance. The detailed annotation of the hemocyte transcriptome will uncover new facets of hemocyte development and function in adult dipterans and is a valuable community resource for future studies on mosquito cellular immunity.

**Supplementary Information:**

The online version contains supplementary material available at 10.1186/s12864-024-09986-6.

## Background

*Anopheles gambiae* mosquitoes are very efficient vectors of *Plasmodium falciparum* malaria, the most virulent form of the disease in humans, that resulted in more than 600,000 deaths in 2021 [1]. *A. gambiae* mounts an effective defense response to *Plasmodium berghei* (murine malaria) that limits parasite survival and requires the coordinated activation of epithelial, cellular, and humoral immune responses. *Plasmodium* fertilization occurs in the midgut lumen, giving rise to a motile ookinete stage that must traverse the midgut. Ookinetes cause irreversible damage to the cells they invade [2–4] and trigger the release of Prostaglandin E2 (PGE2), which attracts hemocytes to the midgut [5, 6]. Damaged cells undergoing apoptosis activate a strong nitration response [7], and hemocytes patrolling the basal surface of the midgut release microvesicles when they encounter a nitrated area [5]. The release of hemocyte-derived microvesicles is essential for the effective activation of the mosquito complement-like system [5], which forms a protein complex that binds to the ookinete and lyses the parasite [4].

Based on their morphology, hemocytes are categorized into three subtypes: prohemocytes, oenocytoids, and granulocytes [8]. Prohemocytes are the smallest (5–7 μm in diameter); the most abundant cell type (approximately 70–80% of the total population) and are thought to be the precursors to the other two cell types. Oenocytoids are larger (about 8–15 μm), represent approximately 15–25% of hemocytes, and are involved in pathogen melanization; while granulocytes are the largest (20–25 μm) and the least abundant (2–3%) cell type, and play an important role in eliminating pathogens by phagocytosis. There is also strong evidence that a previous infection with *Plasmodium* boosts the ability of mosquitoes to respond to subsequent infections and granulocytes are key effectors of this enhanced response [9].

Hemocytes are key effectors of mosquito immunity that comprise a very low percentage of the total tissue of an adult female mosquito. Although previous studies explored the differential expression of hemocyte transcripts in *Anopheles stephensi, Anopheles culicifacies* and *Anopheles gambiae* [10–12], a comprehensive identification and characterization of *A. gambiae* hemocyte-specific mRNA transcripts is still lacking. Here we report a novel in-depth hemocyte transcriptome annotation which is built using extensive bulk sequencing of RNA isolated from hemocytes of *A. gambiae* female mosquitoes, with high coverage to detect transcripts present in low abundance. A previous molecular atlas of *A. gambiae* hemocytes using single-cell RNA-seq data analysis confirmed that prohemocytes give rise to granulocytes, which further differentiate into three final effector cells: dividing granulocytes, megacytes and antimicrobial granulocytes [13]. However, it was not possible to determine the developmental lineage of oenocytoids when the RNA transcripts predicted based on the canonical genome were used as a reference in the analysis [13]. Here, we determined the oenocytoid lineage by reanalyzing the previous single-cell RNA-seq dataset using our novel hemocyte transcriptome as a reference.

## Results

### Genome-guided alignment and analysis of Illumina and PacBio sequences

RNA was extracted from hemocytes of *A. gambiae* females collected by perfusion and used to build cDNA libraries for RNA sequencing. The libraries were sequenced with the Illumina platform, which provides an extensive depth of coverage, and with the PacBio platform, which provides long reads that facilitate the correct assembly of isoforms from the same gene generated by differential RNA splicing. A total of 2.7 billion high-quality raw reads were generated with Illumina sequencing, with an average size of 100 bp; while 9.5 million high-quality long reads were obtained with the PacBio sequencing platform with an average size of about 1.7Kb.

**Fig. 1:**
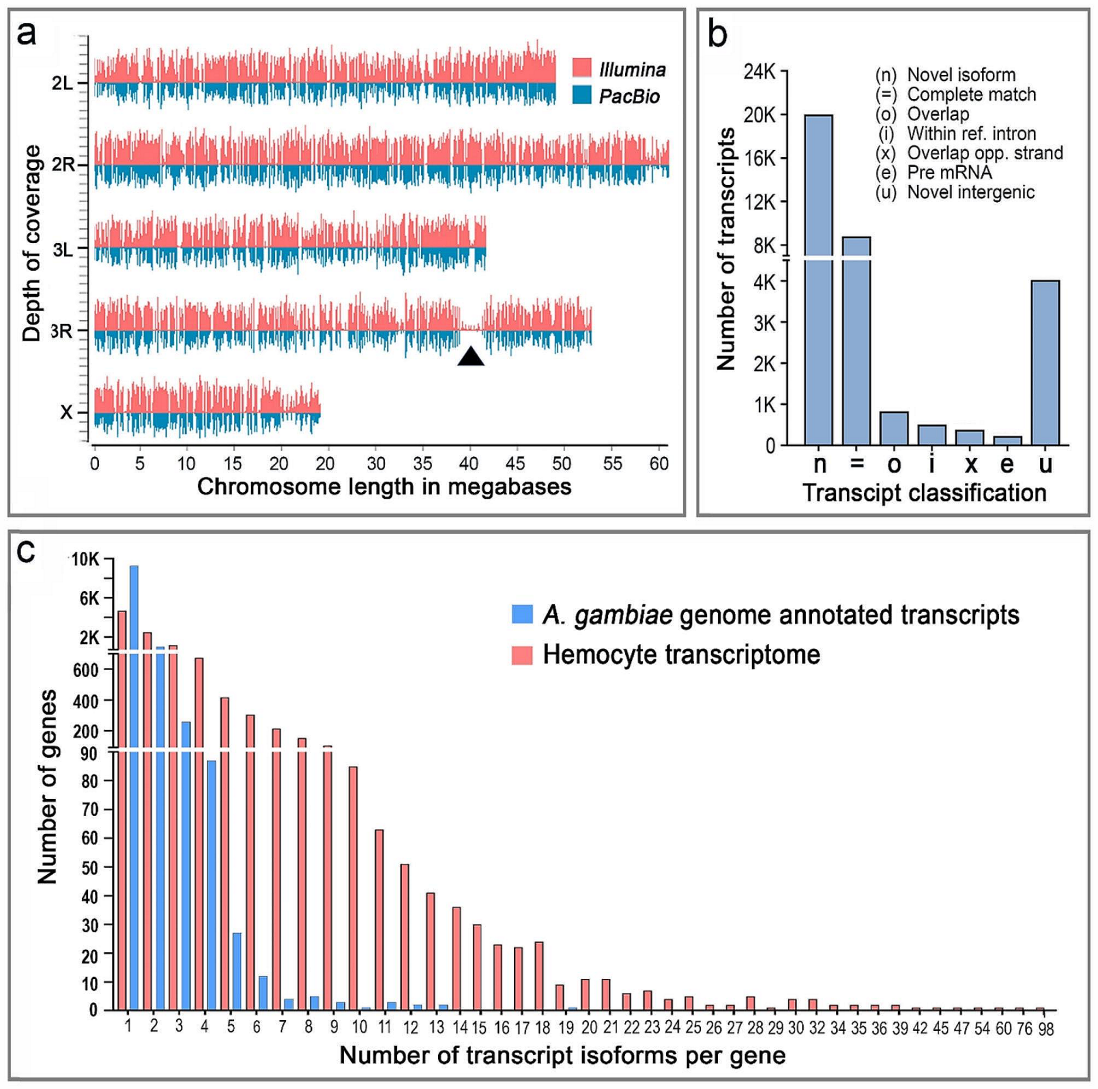
Genome-guided alignment and analysis of Illumina and PacBio sequences. **(a)** Depth of coverage of Illumina (pink) and PacBio (blue) reads mapped to the *A. gambiae* genome. The arrowhead indicates a heterochromatic region in 3R with low read coverage. The average coverage depth of Illumina is 603.38 and PacBio is 46.17. **(b)** Number of transcripts from each structural class identified in the hemocyte transcriptome. **(c)** Distribution of the number of isoforms assigned per gene based on the *A. gambiae* P 4.14 transcript annotation (blue) and the in-depth hemocyte transcriptome (pink)

High-quality reads were mapped to the P4.14 version of *A. gambiae* PEST genome using the HISAT2 and Minimap2 alignment tools for short and long reads, respectively. On average, 90% of the reads from each Illumina library and 99% of the reads from each PacBio library mapped to the *A. gambiae* genome (Table S1), and only the mapped reads were used for transcript assembly. In general, the reads obtained with both sequencing methods were distributed evenly throughout the genome (Fig. 1a), with only a prominent gap in chromosome 3R devoid of reads (see arrowhead in Fig. 1a), corresponding to a region of heterochromatin that has been previously documented [14]. Some small gaps are also expected because, presumably, there are a substantial number of genes in adult females that are not expressed in hemocytes.

Transcript models were constructed with a hybrid methodology that combined Illumina and PacBio genome-mapped reads, using the StringTie2 transcriptome assembler guided by *A. gambiae* PEST P4.14 gene annotations [15]. Transcripts exhibiting an identity of 98% or higher to longer transcripts were subsequently excluded, to minimize the inclusion of partial transcripts. As a result, a hemocyte transcriptome was established, consisting of 34,939 distinct transcripts. The transcripts models in the hemocyte transcriptome were compared with the predicted mRNAs of the current *A. gambiae* PEST P 4.14 genome annotation using the GffCompare utility and were further categorized according to their structural features. The structural classification of the transcripts and their relative abundance is illustrated in Fig. 1b. In general, 8,780 transcripts (25.12%) have a complete match to previously annotated transcripts predicted from the genome sequence (“=”), while the remaining 26,000 transcripts (74.41%) are potential novel transcripts. The majority of novel transcripts (20,008 of 26,000; 76.95%) represent novel isoforms (“n”) of transcripts from previously annotated genes (Fig. 1b). These novel transcripts had several structural properties that are common to alternatively spliced genes, such as exons that match to a reference transcript, but had different lengths (alternative splice donor or acceptor usage), inclusion of all or some introns between multiple exons (intron retention), or combinations of different exons (mutually exclusive exons). A total of 4,020 transcripts (11.5% of the total) were mapped to the intergenic regions unknown to code for transcripts (“u”), corresponding to novel genes discovered in this hemocyte transcriptome annotation. A few transcripts (831; 2.37% of total) overlap (“o”) with reference exons or contain reference exons within their intron(s) and 514 transcripts (1.47% of total) were fully contained in the intron of a reference transcript (“i”). A small fraction (382, 1.09% of total) of novel transcripts had exons overlapping with the opposite strand of a known reference transcript (“x”), and 234 transcripts (0.67% of total) contained an exon that partially mapped to a known intron and, thus, could be part of pre-mRNA (“e”). A few transcripts (159, 0.45%) were designated as “polymerase run-ons” because they were close to a known transcript but had no direct overlap with it, and only 11 transcripts (0.03%) were designated as read mapping errors (not shown in the figure).

Among the 34,939 identified hemocyte transcripts, 84% (29,235) were assigned to 11,427 existing AGAP gene model IDs during StringTie2 assembly. The fact that many transcripts were assigned an AGAP ID suggested that many novel transcript variants (isoforms) per gene were present in the hemocyte transcriptome assembly that has not been identified in the latest genome annotation (version P4.14). Indeed, while a maximum of 20 transcripts per gene were annotated in the *A. gambiae* genome (version P4.14), we identified genes with up to 98 isoforms resulting from differential mRNA splicing (Fig. 1C and Fig. S1). Moreover, we found more genes with multiple isoforms assigned to them in the hemocyte transcriptome (Fig. 1c). For example, while only 42 genes in the *A. gambiae* genome reference have five or more isoforms (five to 20 per gene), we identified 1,665 genes with five or more isoforms (five to 98 per gene) in the hemocyte transcriptome assembly (Fig. 1c, Fig. S1 and Additional file 1). Overall, 5,666 genes have a higher number of transcript isoforms annotated in the hemocyte transcriptome than in the transcriptome predicted from the reference genome (Fig. 1c and Fig. S1).

### Features of annotated genes with a large number of isoforms

We were intrigued by the abundance of genes with a large number of isoforms in our transcriptome. The functional categories of the predicted proteins encoded by genes with multiple isoforms were investigated to gain some insight into the potential functional significance of these hemocyte-expressed genes with many different mRNA variants. The five most abundant functional categories of proteins coding genes with five or more isoforms were protein kinases (66 genes with five to 26 isoforms), Zinc finger domains proteins (22 genes with five to 98 isoforms), E3 ubiquitin ligases (20 genes with five to 13 isoforms) and proteins with RNA Recognition Motifs (RRM) (12 genes with one to nine isoforms). The full list of all functional categories is indicated in Table S2.

### Circular RNA transcripts

The Sua Illumina RNA-seq dataset (Sua Naïve and Challenge samples) was generated using total RNA (without poly-A selection) as a template, making it possible to explore the potential presence of circular RNAs (circRNA). The circRNA analysis toolset of the CIRCexplorer2 software, which annotates back-splicing junction reads with user-provided gene annotations, was used to analyze the Sua Illumina RNA-seq dataset, using both the Vectorbase P4.14 version annotated transcripts of the *A. gambiae* PEST genome and the novel hemocyte transcriptome annotation. However, the software failed to detect any fusion junctions, indicative of a lack of circRNA transcripts.

### Features of noncoding and protein-coding transcripts

**Fig. 2:**
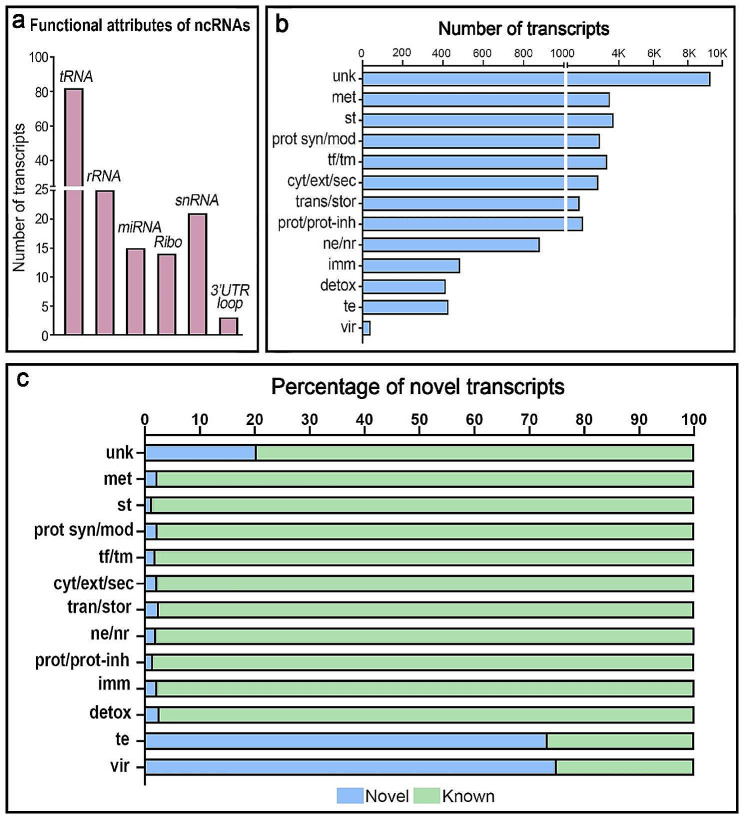
Functional classification of the hemocyte mRNA transcripts. **(a)** Number of transcripts per functional class of noncoding RNAs identified in hemocyte transcriptome. **(b)** Number of transcripts in the hemocyte transcriptome coding for proteins from different functional categories. **(c)** Percentage of novel intergenic (blue) and known (annotated with an AGAP ID, green) transcripts identified in the hemocyte transcriptome for each functional category. (**unk**: unknown, **met**: metabolism, **st**: signal transduction, **prot syn/mod**: protein synthesis/modification, **tf/tm**: transcription factor/transcription machinery, **cyt/ext/sec**: cytoskeleton/extracellular matrix and adhesion/secreted, **tran/stor**: transport/storage, **ne/nr**: nuclear export/nuclear regulation, **prot/prot-inh**: protease/protease inhibitor, **imm**: immunity, **detox**: detoxification and oxidant metabolism, **te**: transposable element, **vir**: viral product)

The protein-coding potential of all transcripts (34,939) was analyzed using CodAn and Transdecoder software and 89.8% of transcripts were predicted to be protein coding and 10.2% to be noncoding RNAs (ncRNAs). The number of ncRNAs identified (3,561) is substantially higher than the 738 ncRNAs that had been previously predicted by the *A. gambiae* genome annotation. We were able to assign distinct functional attributes to 160 ncRNAs (4.5%) using the Rfam database of ncRNA families. Most of these transcripts (51%) were designated as tRNAs (Fig. 2a), while others corresponded to ribosomal RNAs (rRNAs) (16%), microRNAs (9%), ribozymes (9%), different types of small nuclear RNAs that are part of the spliceosome complex (13%) and histone 3’UTR stem-loops (2%). A small percentage (5.5%) of ncRNA transcripts that appear to result from polymerase run-on, potential mapping error, or pre-mRNA transcripts were excluded from the analysis. Novel ncRNAs without any identifiable structural attribute (3203/3561, 90%) were designated as long noncoding RNAs (lncRNAs) and they ranged in length from 200 to 7354 nucleotides (Supplementary information: Annotation table of hemocyte transcripts).

### Functional classification of protein-coding transcripts

The functional class of the 28,697 proteins encoded by hemocyte transcripts was established by BLAST analysis of the predicted peptide sequences against 11 databases, including the annotated *A. gambiae* genome. The detailed annotation table is provided in Supplementary information (Annotation table of hemocyte transcripts). The function of 32.4% of proteins is unknown, and this class encompasses transcripts that exhibit negligible similarity to annotated proteins or bear resemblance to proteins of unidentified function. Their overall high prevalence reflects the large number of mosquito proteins whose function remains to be established. The other most abundant functional protein classes are signal transduction (12.8%), metabolism (12.1%), transcription machinery (11.5%), and protein synthesis and modification (10.1%) (Fig. 2b).

Approximately 91% of the protein-coding transcripts (28,697 transcripts) mapped to previously annotated genes with a corresponding AGAP ID, and represented protein variants from differentially spliced mRNAs, while 2,678 (approximately 9%) were potential novel protein-coding transcripts that did not map annotated genes. For most functional classes, only 2–3% of the peptides were novel (Fig. 2c). However, there was a higher proportion of novel proteins for those with unknown function (20.4%), for proteins of viral origin (75%) and transposable element proteins (73.3%) (Fig. 2c). Of the proteins related to transposable elements, 28.5% were reverse transcriptase (Table S3); and 37.3% of the immune-related proteins expressed in hemocytes contain domains involved in pathogen recognition or immune response such as immunoglobulin, ficolin and lectin domains. Most novel mRNAs predicted to code for secreted proteins with a predicted signal peptide (SigP) (62.9%) also have a stop codon, suggesting that they encode for full-length peptides ranging in size from 101 to 183 amino acids (Fig. S3).

### Hemocyte single-cell transcriptome clustering and lineage analysis

An *A. gambiae* hemocyte cell atlas was previously established using single-cell RNA-seq analysis of the transcriptome of approximately 5,300 individual cells using the predicted transcripts from the annotation of the *A. gambiae* genome (P4.9 version) as reference [13]. The atlas identified the three known major hemocyte types: prohemocytes, oenocytoids, and granulocytes; including two prohemocyte subtypes (PHem1 and PHem2) and three granulocyte subtypes (Gran1, Gran2, and Gran3). In addition, three novel granulocyte effector subpopulations were defined: dividing granulocytes, antimicrobial granulocytes, and a new cell type which was named “megacytes”. These nine hemocyte subpopulations were revealed by graph-based clustering and had their identity classes established through the identification of gene sets (marker genes) uniquely expressed in the nine different clusters [13].

**Fig. 3:**
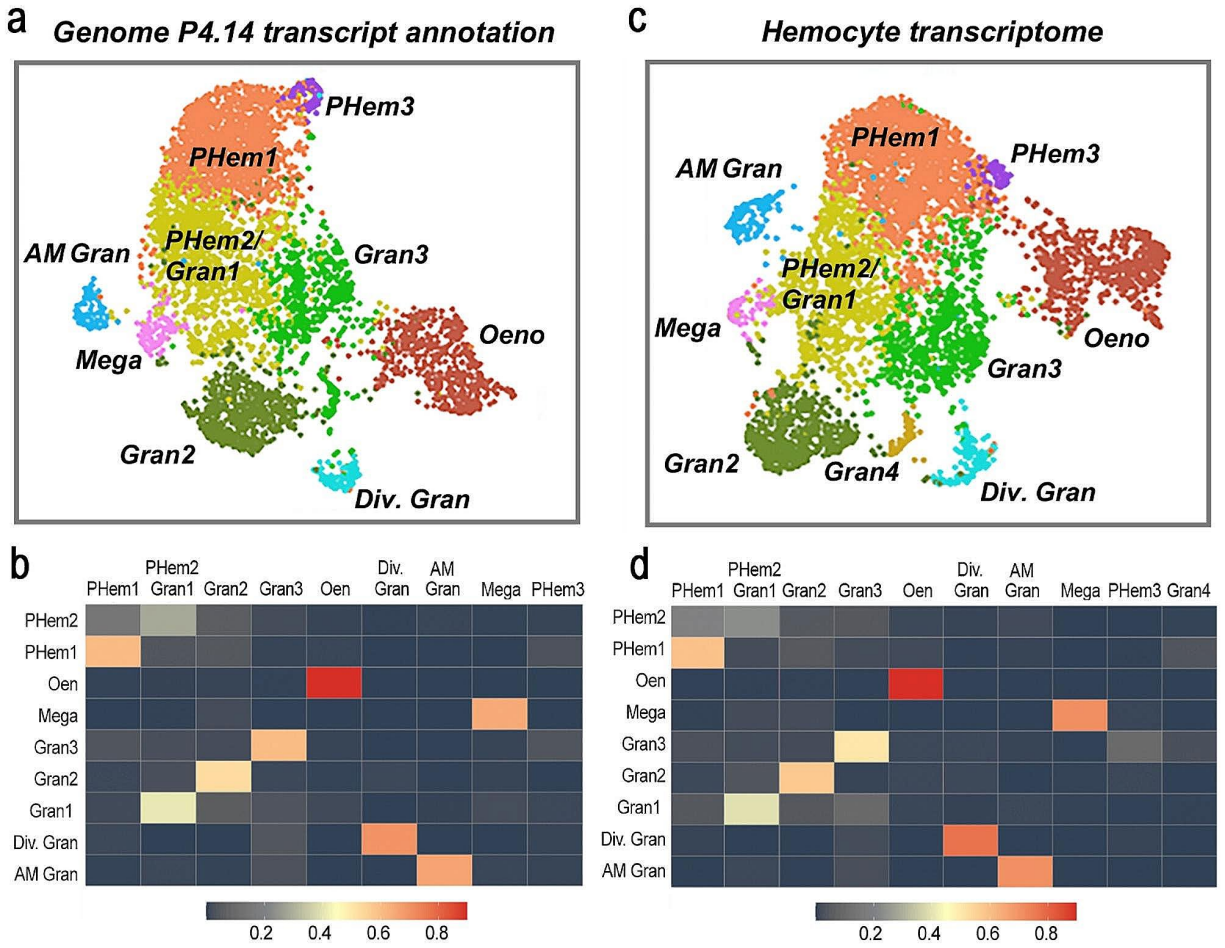
Hemocyte single-cell transcriptome analysis. **(a**) Uniform Manifold Approximation and Projection (UMAP) of single-cell hemocyte transcriptomes clustered by for Seurat analysis using the annotated transcripts based on the *A. gambiae* genome (P 4.14) as reference. Each cluster corresponding to a different hemocyte type is shown with a different color. **(b)** Jaccard plot showing the similarity between the new hemocyte clusters (horizontal list) and the hemocyte subpopulations previously reported (vertical list). **(c)** UMAP of single cell hemocyte transcriptomes clustered by for Seurat analysis using the hemocyte transcriptome as reference. Each cluster corresponding to a different hemocyte type is shown with a different color. **(d)** Jaccard plot showing the similarity between the new hemocyte clusters (horizontal list) and the hemocyte subpopulations previously reported (vertical list)

We re-analyzed the same data set using either the predicted transcripts based on the most recent annotation of the *A. gambiae* genome (P4.14 version) or our high-resolution hemocyte transcriptome as a reference and compared the results of the clustering and lineage analysis using these two different transcript references. A total of nine hemocyte clusters were obtained using the transcripts predicted in the P 4.14 version of the *A. gambiae* genome (Fig. 3a). The similarity of these clusters with the previously defined hemocyte types was determined by calculating the Jaccard index. Based on the Jaccard similarity index (Fig. 3b), all the nine previously described hemocyte clusters were also present with two minor differences: the previously defined PHem2 and Gran1 clusters merged into a single cluster (PHem2/Gran1) (Fig. 3a-b), and a new small prohemocyte cluster was identified. This new cluster was named PHem3 (Fig. 3a-b) because it does not have a strong Jaccard similarity index with any of the PHem clusters previously reported.

Ten hemocyte clusters were defined using the high-resolution hemocyte transcriptome as a reference (Fig. 3c). Clusters with high Jaccard similarity to all hemocyte types previously reported were identified, which also included the PHem2/Gran1 and the PHem3 clusters (Fig. 3c-d), as well as an additional small granulocyte cluster that was named Gran4 (Fig. 3c-d), based on a modest Jaccard index similarity to Gran3 hemocytes. Several markers that define the clusters are novel transcripts identified in the hemocyte transcriptome (two to six novel markers per cluster) (Fig. S2) implying their potential contribution to the refined cell clustering. The complete list of genes that define the different hemocyte clusters using the hemocyte transcriptome as references is provided in Additional file 2.

### Functional differences between hemocyte subpopulations

Potential functional differences between hemocyte subpopulations were explored by comparing the relative abundance of different functional protein classes encoded by the marker transcripts that define the hemocyte clusters. The functional class of the proteins encoded by the marker genes was established based on the detailed annotation table of hemocyte transcripts (Supplementary information -Annotation table of hemocyte transcripts). The relative abundance of the different functional classes in each hemocyte cluster revealed some striking differences (Fig. S4). The PHem3 cluster has the highest proportion (39%) of genes involved in energy metabolism, suggesting that these cells are metabolically more active than other hemocytes. Oenocytoids, in turn, have a higher proportion (about 16%) of marker genes involved in protein transport and storage, possibly related to the synthesis of proteins involved in melanization, such as prophenoloxidases; while both AM granulocytes and oenocytoids exhibit a higher proportion (about 9%) of immune-related genes than other clusters, suggesting that their antimicrobial response involves synthesis and secretion of immune effectors peptides/proteins. Finally, the proportion of genes involved in ROS detoxification is higher in the Gran2 and Gran4 clusters (10% and 11.11% respectively). These are genes that maintain the redox state of the cell and are known to regulate the proliferation of mammalian cells and the function of immune cells [16, 17].

The functional differences between hemocyte subtypes were further investigated by subjecting the list of marker genes from each cluster to Gene Ontology (GO) Enrichment Analysis for biological processes, molecular function, and cellular components. The summary of the list of marker genes with significant enrichment in each cluster is provided as Additional file 3 (GO enrichment analysis HC markers). We found that the markers for the PHem3 cluster are enriched for genes involved in glycolysis, while the markers of the PHem1 cluster are enriched for genes involved in aerobic respiration. The genes involved in carboxylic acid metabolism and zinc ion transport are predominant in the oenocytoid cluster markers and the PHem2/Gran1 cluster markers are enriched with lysosome-related genes and genes involved in cytoplasmic vesicle traffic. The genes regulating mRNA splicing and decay are prominent in the AM granulocyte cluster markers and genes that are part of the endomembrane system and vesicle transport is dominant amongst the markers of the megacyte cluster. The Gran 3 cluster markers are enriched for genes related nucleotide metabolism in addition to protein glycosylation and secretion. Genes functioning in extracellular matrix organization are prominent in Gran 2 cluster markers, whereas Gran 4 cluster is enriched for genes involved in cytoskeleton and glutathione metabolism. Finally, the markers of the dividing granulocyte cluster show enrichment of genes involved in active cell division.

### Hemocyte lineage analysis

Prohemocytes are thought to be the precursors of other hemocytes [18–21], and a previous hemocyte lineage analysis, based on single-cell transcriptome analysis, defined a clear differentiation pathway in which prohemocytes gave rise to the granulocyte lineage [13]. This is a sequential process in which the PHem1 subpopulation gave rise to PHem2, which are the precursors of Gran1 hemocytes. Some of these cells differentiate into antimicrobial granulocytes, while others give rise to Gran2 and Gran3 hemocytes that further differentiate into megacytes and dividing granulocytes, respectively. However, it was not possible to define the differentiation pathway of oenocytoids [13].

**Fig. 4:**
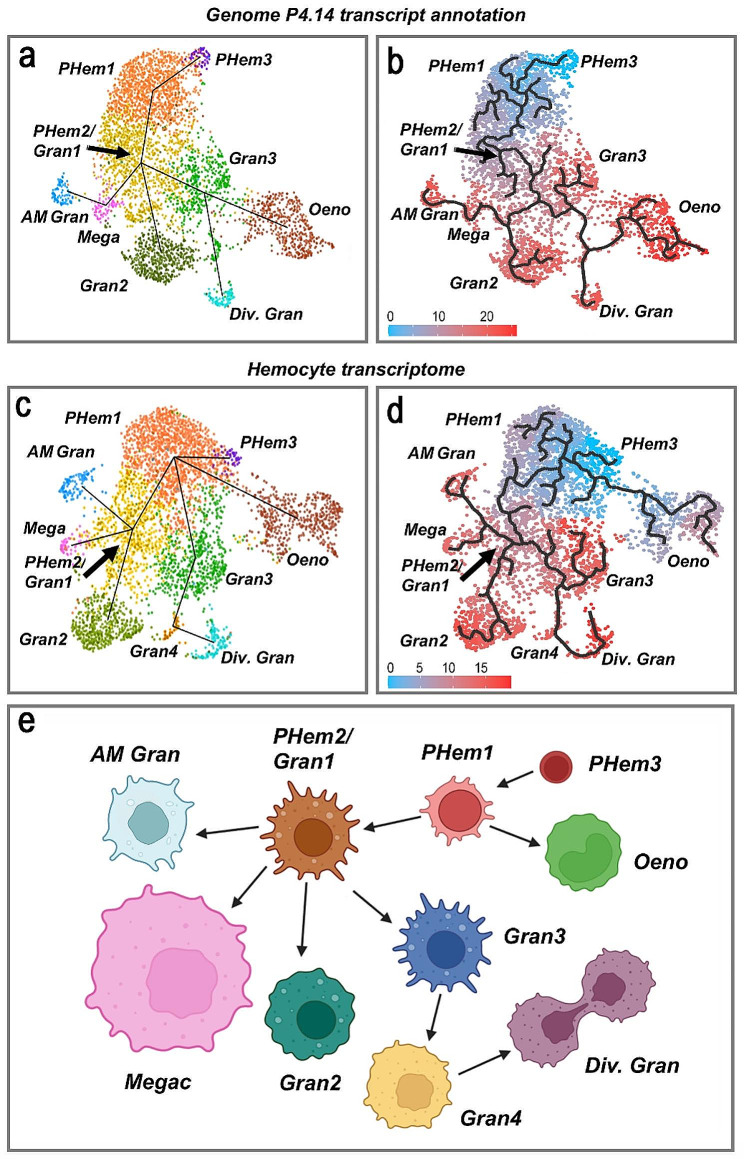
Lineage analysis to determine hemocyte differentiation. **(a)** Lineage of hemocyte clusters defined using the genome (P4.14 version) annotated transcripts as a reference by unsupervised analysis using the TSCAN software. **(b)** Pseudotime plot of the same clusters analyzed with Monocle3, using PHem3 as root. **(c)** Lineage map of hemocyte clusters defined with the hemocyte transcriptome as a reference by unsupervised analysis using the TSCAN software. **(d)** Pseudotime plot of the same clusters with Monocle3, using PHem3 as root. **(e)** Schematic summary of the lineage of hemocyte subpopulations based on the differentiation pathways determined by Moncole3 pseudotime analysis based on hemocyte transcriptome annotation as a reference

The lineage of the hemocyte clusters from the data re-analyzed using the transcripts from the annotated genome (version P 4.14) as reference were subjected to an unsupervised pseudo-temporal ordering (or trajectory inference analysis) based on gradual changes in the transcriptomic profile of hemocytes using the Tools for Single Cell Analysis (TSCAN). TSCAN identified PHem3 as the initial point of differentiation that gives rise to PHem1 (Fig. 4a). The lineage analysis was confirmed using an independent pseudo-temporal ordering method with the Monocle 3 software that defines the changes in gene expression that are part of a dynamic biological process, such as hemocyte differentiation, as a trajectory path and places each cell according to its state in the trajectory (referred to as pseudotime). PHem3 was used as the root in the Monocle 3 analysis, based on the TSCAN software results. Both TSCAN and Monocle 3 predicted that antimicrobial granulocytes, Gran2, and megacytes derive from the PHem2/Gran1 cluster, while Gran3 gives rise to the dividing granulocytes and the oenocytoid lineages (Fig. 4b). It is very unlikely that Gran3 hemocytes differentiate into oenocytoids, because Gran3 hemocytes are cells already committed to the granulocyte lineage, and granulocytes are functionally and morphologically distinct from oenocytoids.

When the in-depth hemocyte transcriptome was used as a reference, the TSCAN software also identified PHem3 as the initial point of differentiation that gives rise to PHem1. However, the predicted lineage is different from that in which the genome annotation was used as a reference because both TSCAN and Monocle 3 predict that PHem1 gives rise to the granulocyte and the oenocytoid lineages (Fig. 4c). The pathway of hemocyte differentiation obtained with Monocle3 (Fig. 4d) predicts that oenocytoids derive directly from PHem1, and PHem1 also gives rise to the PHem2/Gran1 cluster (Fig. 4d-e). Furthermore, the PHem2/Gran1 cells differentiate into four granulocyte clusters: antimicrobial granulocytes, megacytes, Gran2, and Gran3. Gran3, in turn, gives rise to dividing granulocytes, with the new small Gran4 cluster as an intermediate stage of differentiation (Fig. 4d-e).

## Discussion

A combination of a large number of short Illumina reads with long PacBio reads made it possible to achieve deep coverage of the mosquito hemocyte transcriptome and to annotate new genes and novel transcripts from many genes that code for multiple transcripts and isoforms through extensive differential splicing. It would be interesting to further investigate if these novel isoforms also exist in other tissues of adult mosquitoes or are exclusive to hemocytes. The most prominent categories of proteins with many multiple isoforms in hemocytes are protein kinases, zinc finger domain-containing proteins, RNA recognition motif-containing (RRM) proteins, and E3 ubiquitin ligases. Protein kinases are key regulators that bridge different signaling pathways and have diverse functions such as transcriptional regulation, cell proliferation, immune activation, and differentiation of immune cells [22–25]. Previous studies of larval hemocytes of insects such as wax moths, silkworms and fruit flies showed that protein kinases regulate hemocyte motility, adhesion, and phagocytosis in response to bacterial infection or LPS treatment [26–29]. The high diversity of protein kinase isoforms suggests complex and sophisticated regulation of signaling networks in mosquito hemocytes during differentiation and immune response. Zinc finger domain-containing proteins can bind to different nucleic acids such as DNA, ssRNA, dsRNA, and even DNA-RNA hybrids [30–32]. These proteins can recognize specific mRNA motifs and are known to stabilize or regulate the translation of several cytokine mRNAs. Zinc finger proteins regulate hematopoiesis and hemocyte differentiation and are also involved in the immune response of *Drosophila* larval hemocytes to bacterial LPS [33, 34]. In other invertebrates, such as mollusks and shrimps, Zn finger proteins regulate hemocyte hematopoiesis and apoptosis [35, 36]. RRM proteins contain RNA-binding domains that recognize specific sequence elements (such as AU-rich elements) or secondary structural motifs in RNA and regulate mRNA splicing, export, degradation, and translation. In vertebrates, RRM proteins modulate the immune system by regulating the differentiation of immune cells and translation of cytokines, or by resolving inflammation [37–40]. RRM proteins regulate alternative splicing of the Down syndrome cell adhesion molecule (Dscam) in shrimp and crab hemocytes, in response to bacterial and viral infection, and increased production of reactive oxygen species (ROS) and apoptosis in response to viral infection [41–43]. In *Drosophila*, RRM proteins have been shown to regulate hemocyte proliferation, differentiation, and immune response against bacteria [44, 45]. Proteins with Zinc finger and RRM domains could be involved in the post-transcriptional regulation of several mRNAs critical for hemocyte function. E3 ubiquitin ligases catalyze the transfer of ubiquitin to cysteine (Cys) residues of specific protein substrates and play a crucial role in cellular localization, protein stability, and interactions with other proteins. Ubiquitination of specific proteins such as MHC molecules or receptors of signaling pathways is known to modulate immunity in mammals [46, 47]. E3 ubiquitin ligases also maintain the pluripotency of stem cells in mammals [48] and multiple studies in mollusks and shrimps indicate the role of E3 ligase in granulocyte proliferation, inhibition of apoptosis and regulation of immune response against bacteria and autophagy [49–52].E3 ligase could perform similar functions in mosquito hemocytes. Hemocytes are highly plastic cells that can detect different pathogens like bacteria, viruses, and eukaryotic parasites and differentiate into multiple effector cells to mount pathogen-specific immune responses. Thus, the expression of diverse types of protein kinases, zinc fingers, RRMs, and E3 ligases in hemocytes could be crucial for hemocyte plasticity as they respond to infection to efficiently eliminate the different pathogens they encounter.

The two notable categories of hemocyte proteins encoded by novel genes are Reverse transcriptase (RTs) and putative secreted proteins. RTs are multifunctional enzymes with RNA/DNA dependent DNA polymerase activity and RNAseH activity initially identified in single-stranded RNA (ssRNA) viruses that integrate into the host genome. However, there is growing evidence in *Drosophila* and other insects like silkworms, moths, and even mouse embryonic stem cells that show endogenous host RTs may play a major role in antiviral immunity [53–57]. Thus, some of the novel RTs expressed in *A. gambiae* hemocytes might also be involved in antiviral immunity. BLAST analysis of the hypothetical proteins predicted to be secreted by hemocytes with multiple databases indicated that their function is unknown. In other organisms, small signaling proteins, such as cytokines range in size from approximately 50–600 amino acids, while chemokines are slightly smaller (approx., 70–110 amino acids). Even in *Drosophila*, only a few peptides involved in immune signaling, such as Spaetzle and Unpaired, have been characterized. Based on their size and expression in hemocytes, some of these transcripts that contain a signal peptide may encode novel cytokines acting as ligands or activators of receptors from immune signaling cascades.

In addition to new protein-coding genes, 3203 new noncoding genes were identified that possibly function as long noncoding RNAs (lncRNAs). LncRNAs regulate gene expression at multiple levels ranging from epigenetic regulation by chromatin remodeling, to post-translational modification of proteins [58–60]. Recent studies in *Drosophila* show that lncRNAs regulate hemocyte differentiation in larval hemocytes [61], suggesting that some lncRNAs expressed in mosquito hemocytes may also modulate gene expression and regulate the differentiation and function of hemocytes.

The detailed hemocyte transcriptome annotation strengthened and refined the previously published hemocyte atlas and the pathway of hemocyte differentiation in adult *A. gambiae* females. Moreover, the gene ontology enrichment analysis of the hemocyte marker genes helped to gain valuable insights into the development and function of the various hemocytes. Lineage analysis showed that PHem3, a newly defined small prohemocyte cluster, is a precursor cell population that gives rise to prohemocytes. Studies in adult human hematopoietic stem cells and heart stem cells show that stem cell differentiation is associated with drastic metabolic changes and glycolysis is the predominant metabolic state in stem cells, whereas switching to aerobic respiration promotes cell differentiation [62–64]. Thus, the state of energy metabolism may also be important for hemocyte differentiation in *A. gambiae* adult females. The enrichment of marker genes involved in glycolysis in PHem3 cluster and aerobic respiration in PHem1 cluster suggests that PHem3 hemocytes are less differentiated precursors of PHem1 hemocytes, in agreement with the lineage analysis. The identification of hemocyte stem cells in adult stages is still unknown and PHem3 cells may be involved in maintaining and replenishing hemocyte populations. Determining the location, abundance, and proliferation potential of these potential stem cells will be important to establish whether is a hematopoietic organ in adult dipteran insects or if hemocyte stem cells proliferate as free cells in the hemocoel. Our lineage analysis also highlights that using a comprehensive set of mRNA transcripts from the cells under investigation as a reference is essential for a robust single-cell transcriptomic analysis. Several novel genes identified in our in-depth hemocyte transcriptome were important markers of several hemocyte clusters and they made it possible to assign the correct lineage to oenocytoids. Carboxylic acid metabolism mediates melanin synthesis and Zinc is essential for some enzymes involved in melanin biosynthesis [65, 66]. Thus, the enrichment of genes responsible for carboxylic acid metabolism and Zinc ion transport in the oenocytoids cluster may be explained by their role in melanization. The presence of lysosomes is a marker of hemocyte differentiation to granulocytes in marine mollusks [67] and enrichment of lysosome-related genes in the PHem2/Gran1 cluster may represent an early stage of hemocyte differentiation that ultimately gives rise to mature granulocytes. In the crayfish, alternative splicing of the Relish mRNA is important for antimicrobial peptide expression in the gut; and differential splicing of the immunoglobulin-related gene Dscam is observed in response to parasite infection in bumble bees and the defense response of plants against bacterial infection [68–70]. The processing of mRNA transcripts of certain immune genes could be important for the function of AM granulocytes as indicated by the enrichment of genes involved in mRNA splicing in these cells. Because glutathione signaling is known to regulate cell proliferation [17], the enrichment of genes related to glutathione metabolism in the Gran 4 cluster and the lineage analysis, both suggest that they represent an intermediate stage of granulocytes that will give rise to dividing granulocytes, a mitotically active population. The careful annotation of the hemocyte transcriptome, including many novel splice variants and low abundance transcripts, and the detailed description of the predicted function of the proteins they encode are valuable community resources for future studies involving hemocytes and are publicly available at, https://proj-bip-prod-publicread.s3.amazonaws.com/transcriptome/An_gambiae_hemocytes_2022/AgHemocytes.zip.

## Materials and methods

### **Mosquito rearing and*Plasmodium berghei*infection**

The *A. gambiae* G3 strain was reared at 27 °C, 80% humidity on a 12-h light-to-dark cycle. GFP expressing *Plasmodium berghei* strain (ANKA 2.34) was used for mosquito infections and maintained by serial passages in 3- to 4-week old female BALB/c mice or as frozen stocks. Mice with 4–6% parasitemia and two to three exflagellations per field under 400X magnification were used to infect the mosquitoes. The infected mosquitoes were either shifted to nonpermissive (28 °C) temperature immediately after feeding for the Naïve group or maintained at 21 °C for 48 h post feeding and then shifted to 28 °C for the Prime group. For uninfected blood meal, 3- to 4-day old mosquitoes were fed on an anesthetized healthy BALB/c mouse.

### Hemolymph collection

Each mosquito was perfused with 10 µl hemolymph transfer buffer containing 95% Schneider’s media and 5% citrate buffer (modified anticoagulant buffer). For the hemolymph-transfer experiment, each mosquito was bled with 6µL of hemolymph transfer buffer. Hemolymph from 30 donor mosquitoes were pooled, and centrifuged at 6000 rpm for 15 min at 4 °C. The supernatant was collected and stored in aliquots at -80 °C. One hundred and fifty nanoliters of cell-free supernatant was injected into each mosquito.

### Total RNA isolation from hemocytes

Ten microliters of hemolymph was perfused from each mosquito using 95% Schneider’s media and 5% citrate buffer (modified anticoagulant buffer) and added directly to a tube containing 750µL TRIzol LS reagent. Twenty mosquitoes were pooled in each sample to isolate RNA. Two hundred microliters of chloroform was added to each tube and vigorously shaken for 15 s. The solution was added to Phasemaker tubes (Invitrogen A33248, prespun at 1,200 g for 2 min) and centrifuged at 12,000 g for 15 min at 4 °C to separate the aqueous and organic phases. After collecting the aqueous phase, Linear acrylamide (20 µg/mL, Thermo Fischer Scientific AM9520) was added to each tube and mixed well. Five hundred microliters of Isopropanol was added to each tube, mixed by inverting the tubes, and incubated at RT for 45 min to precipitate the RNA. The tube was centrifuged at 12,000 g for 15 min at 4 °C. The RNA pellet was washed twice with 1mL 75% ethanol and dissolved in 20µL nuclease-free water post drying. The integrity of RNA was checked using the Agilent Tapestation 4200 instrument before library preparation.

### Sample information

RNA was isolated from hemocytes collected from mosquitoes subjected to different treatments. Mosquitoes were either injected with dsRNA against Cactus or LacZ (control), and hemocytes were collected 4 days post injection and allowed to attach on a glass surface at 4 °C for 30 min (bound) and the remaining fraction (unbound) [71]. Mosquitoes were either primed [9] with *P. berghei* and hemocytes were collected 6 days post priming from control NP_Naive and NP_Prime samples. Mosquitoes were injected with cell-free hemolymph from naturally primed (HDF_Prime) or Control Naïve (HDF_Naïve) mosquitoes 48 h post blood meal from a healthy mouse and hemocytes were collected 6 days post injection. Mosquitoes were injected with 150 nL Sua cell supernatant pre-treated with or without *E. coli* acetone powder and arachidonic acid 48 h post blood meal from a healthy mouse and hemocytes were collected 2, 4, and 6 days post injection (Sua_Naive and Sua_Challenge).

### Illumina sequencing

Total RNA was used to generate inverse rRNA-selected RNA sequencing libraries for the Sua Naïve and challenge samples. The bulk RNAseq libraries were created using TruSeq Stranded Total RNA LP Gold kit and the libraries were pooled and sequenced (paired end) using Illumina Novaseq 6000 instrument. For the ds LacZ and ds Cactus samples, Poly-A selected RNA sequencing libraries were created using NEBNext Ultra II Directional RNA Kit with Sanger Unique Dual Indexes and Kapa Hifi polymerase and sequenced paired end on Illumina HiSeq 4000 instrument.

### PacBio sequencing

For PacBio sequencing, Iso-Seq libraries were created using SMRTbell prep kit 3.0 with cDNA oligo dT selection and sequenced on the PacBio Sequel II instrument.

### Transcriptome assembly and transcript identification

#### P4.14 Illumina and PacBio sequencing reads

Illumina sequencing reads were preprocessed using TrimGalore v0.6.6 to ensure sequence adapter removal and to filter out low-quality (Phred score < 30) or short (less than half the target sequencing length) reads [72]. High-quality reads were then aligned to the P4.14 version of the *A. gambiae* PEST genome using HISAT2 v2.2.1 using “downstream-transcriptome-assembly” mode [73]. PacBio Circular Consensus Sequencing reads were also aligned to the *A. gambiae* PEST genome using MiniMap2 v2.17 while in “splice:hq” mode [74]. Subsequently, Illumina and PacBio read alignments were used jointly for transcript assembly using StringTie2 v2.2.1 in “mix” mode, while guided by existing *A. gambiae* PEST P4.14 transcript annotations [15]. Next, putative truncated transcripts, characterized by at least 98% identity to nearby transcripts, were identified by using CD-HIT-EST and removed [75]. Lastly, single-exon transcripts without a reported strand were removed to avoid ambiguity.

#### Prediction of known and novel protein-coding transcripts and coding DNA sequences

Transcript type class (=, c, k, m, n, j, e, o, s, x, I, y p, r, u) and association with genetic locus were predicted by comparison of the assembled hemocyte transcripts to existing *A. gambiae* PEST P4.14 transcripts using GffCompare v0.11.6 [76]. Known transcripts were those denoted by exact match (=) to the reference, whereas novel protein-coding transcripts could be of other types. Protein-coding DNA sequences (CDs) and 5’ UTR and 3’UTR were predicted first using CodAn using a probabilistic generalized hidden Markov model which permitted CDS detection of both full-length and partial/fragmented CDSs using parameters pretrained on invertebrate protein-coding genes and genomes [40]. Transcripts were also run through Transdecoder to detect further ORFs and 5’/3’ UTRs using homology search as ORF retention criteria (BLASTP matches to UniProtKB/Swiss-Prot database and HMMER matches to InterPro PFAM-A database). These predicted CDS, 5’ and 3’ UTR sequences were compiled for further functional annotation.

### Functional annotation of CDS

Functional annotation of the extracted CDS was performed by an in-house program that scans a vocabulary of approximately 400 words and their order of appearance in the protein matches from BLASTp and rpsBLAST results against different databases (Transcriptome Shotgun Assembly, a subset of the Non-Redundant, Refseq-Invertebrate, Refseq-Vertebrate, Refseq-Protozoa, *An. Gambiae* genome, UNIPROT, CDD, SMART, MEROPS and PFAM), including their e-value and coverage. The final annotated CDS and the ncRNA were exported to a Windows-compatible hyperlinked Excel file and are available for download (Supplementary information).

### Noncoding RNA detection and annotation

Transcripts that were not predicted as protein coding by either CodAn (v1.1.0) or Transdecoder were investigated for potential ncRNA identification according to several criteria [72]. First, transcripts greater than 200nt with assigned Stringtie classes likely to correspond to potential ncRNAs were selected (u, i, x, as well as j, k, y, n, m, c, o). We removed transcripts with e, p, and s classes from consideration. Detection of identifiable short ncRNA types was performed using INFERNAL v1.1.4 with the Rfam database [77]. Finally, the remaining ncRNA transcripts were denoted as potential long noncoding RNAs.

### scRNAseq data analysis

10x Genomics single-cell sequencing data of *A. gambiae* hemocytes was downloaded (ArrayExpress accession number E-MTAB-9240) and analyzed as previously described [13]. CellRanger v7.0.0 was used for aligning sequencing reads to the *A. gambiae* PEST P4.14 reference genome and to generate two distinct sets of gene counts (feature-barcode matrices). One set was generated utilizing the transcript annotations from the *P4.14* reference, and the other set by utilizing the new hemocyte transcriptome annotations. Provided cell annotations [78] were then used to filter for only the hemocytes comprising the final scRNA-Seq atlas. Seurat v4.3.0.1 was used for subsequent preprocessing and integration procedures in parallel analyses of the two sets of gene counts. First, the raw expression data of each sample were subjected to log-normalization using the “NormalizeData” function. Subsequently, the top 2000 most variable genes were independently identified for each sample using the “FindVariableFeatures” function and tested for suitability as integration anchors using the “FindIntegrationAnchors” function. These anchors were then used in the “IntegrateData” function to integrate the samples into a single dataset. Following integration, the dataset was scaled using the “ScaleData” function, and a principal component analysis was conducted using the “RunPCA” function. A Uniform Manifold Approximation and Projection (UMAP) representation of the scaled integrated dataset was then constructed using the “RunUMAP” function, using the top 20 Principal Components. Clusters were then identified using the `FindNeighbors` and `FindClusters` functions with a resolution parameter of 0.4.

Trajectory inference analysis was then performed on the two single-cell UMAP projections (P4.14 and hemocyte versions) using two distinct inference methodologies, Tools for Single Cell Analysis (TSCAN) and Monocle3 [79, 80]. TSCAN analyses were performed using the “quickPseudotime” function without a trajectory starting point (or set of root cells) defined, which generated a minimum spanning tree based on distances between mutual nearest neighbors. Monocle3 analyses were performed using the default parameters of the “learn_graph” and “order_cells” function with the PHem3 cell cluster used as the set of root cells. This resulted in a suitable principal graph for each projection and an estimation of the pseudotime ordering of the cells.

To perform functional analysis of the marker genes of each hemocyte cluster provided in the Additional file 2 (Marker for each hemocyte cluster), the functional classification of each marker gene was assessed in accordance with the Annotation table of hemocyte transcripts provided in the Supplementary information. In addition, Gene Ontology Enrichment analysis for the marker genes for each hemocyte cluster was performed using the *A. gambiae* reference list of all genes provided on the website database based on Panther Overrepresentation Test (Released 20,231,017) with the GO Ontology database DOI: 10.5281/zenodo.7942786 Released 2023-01-05 as the annotation version and release date (https://geneontology.org/) [81–83]. The enriched “GO Biological process complete”, “GO Molecular function complete” and “GO Cellular component complete” were analyzed including Fisher’s exact test, and only the significant results were considered (calculated False Discovery Rate, FDR *P* < 0.05) for interpreting the results.

### Electronic supplementary material

Below is the link to the electronic supplementary material.

Supplementary Material 1

## Data Availability

The transcriptome data was deposited to the National Center for Biotechnology Information (NCBI) under Bioproject PRJNA1010716 and Biosamples accession SAMN37195635 - SAMN37195684. The raw reads were deposited to the Sequence Reads Archive of the NCBI under accession SRR25788630 - SRR25788677 and the unique CDS were deposited to the Transcriptome Shotgun Assembly (TSA) under accession GKOR01000000. Furthermore, the hyperlinked Excel file containing the functional annotation of the putative CDS can be downloaded from the link, https://proj-bip-prod-publicread.s3.amazonaws.com/transcriptome/An_gambiae_hemocytes_2022/AgHemocytes.zip.
