# Supplementary material for: In-depth transcriptomic analysis of Anopheles gambiae hemocytes uncovers novel genes and the oenocytoid developmental lineage
Source: BMC Genomics. 2024 Jan 19;25:80. doi: 10.1186/s12864-024-09986-6 (PMC10799387; doi:10.1186/s12864-024-09986-6)
Supplement: Supplementary file 1 — Supplementary Material 1 [file 12864_2024_9986_MOESM1_ESM.docx]

**Supplementary Information**

**In-depth transcriptomic analysis of *Anopheles gambiae* hemocytes uncovers novel genes and the oenocytoid developmental lineage**

Banhisikha Saha^1^**,** Colton M McNinch^2^, Stephen Lu^3,^ Margaret C.W. Ho^2^, Stephanie Serafim De Carvalho^1^, and Carolina Barillas-Mury^1*^

^1^Laboratory of Malaria and Vector Research, National Institute of Allergy and Infectious Diseases, National Institutes of Health, Rockville, United States.

^2^Bioinformatics and Computational Biosciences Branch, Office of Cyber Infrastructure and Computational Biology, National Institute of Allergy and Infectious Diseases (NIAID), National Institutes of Health, Bethesda, MD 20892, USA

^3^Vector Biology Section, Laboratory of Malaria and Vector Research, National Institute of Allergy and Infectious Diseases, Bethesda, MD, USA

^*^ Corresponding author, email: cbarillas@niaid.nih.gov

**Supplementary Figures**


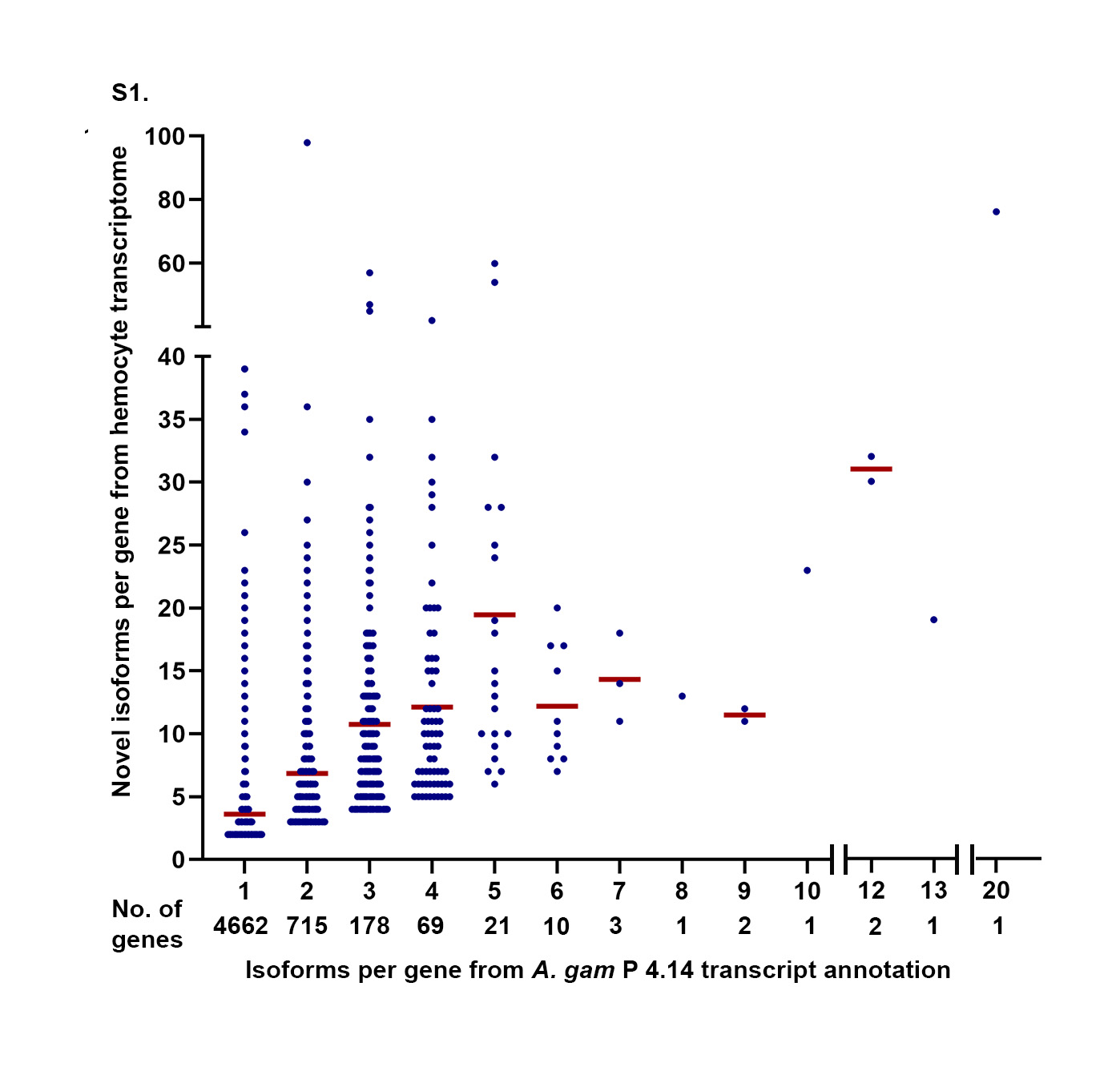


**Fig. S1** Novel isoforms per gene identified in hemocyte transcriptome.

Graph depicting the range of novel isoforms per gene detected in the hemocyte transcriptome for genes with specific no. of isoforms assigned in the *A. gam* P 4.14 transcript annotation.


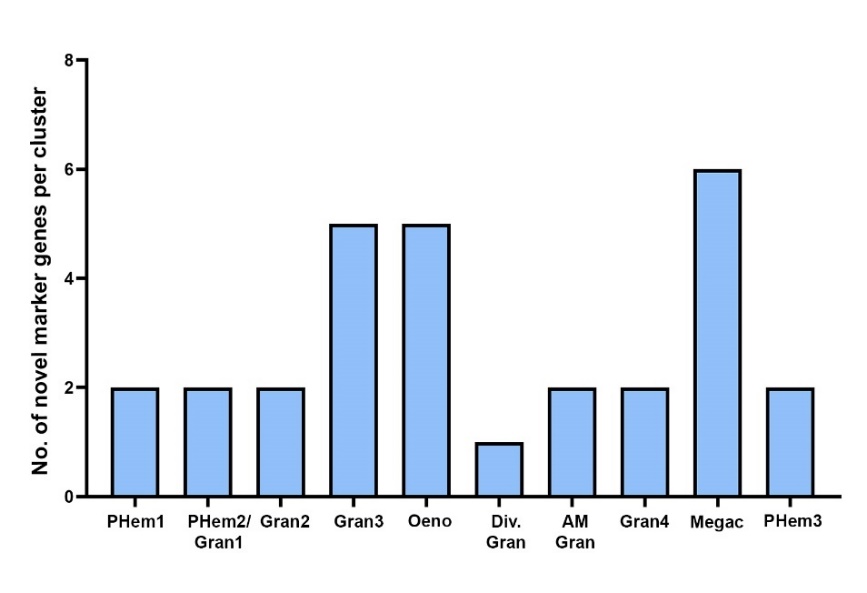


**Fig. S2** Novel gene identified as hemocyte cluster marker.

Graph showing the number of novel genes identified as marker in each hemocyte cluster using hemocyte transcriptome as reference.


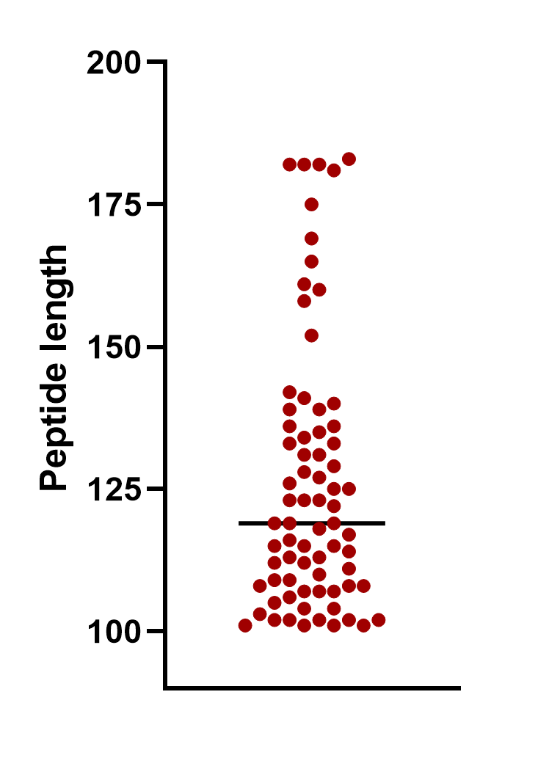


**Fig. S3** Length of novel secreted peptides.

Graph showing the length distribution with median length of novel putative secreted peptides in the hemocyte transcriptome.


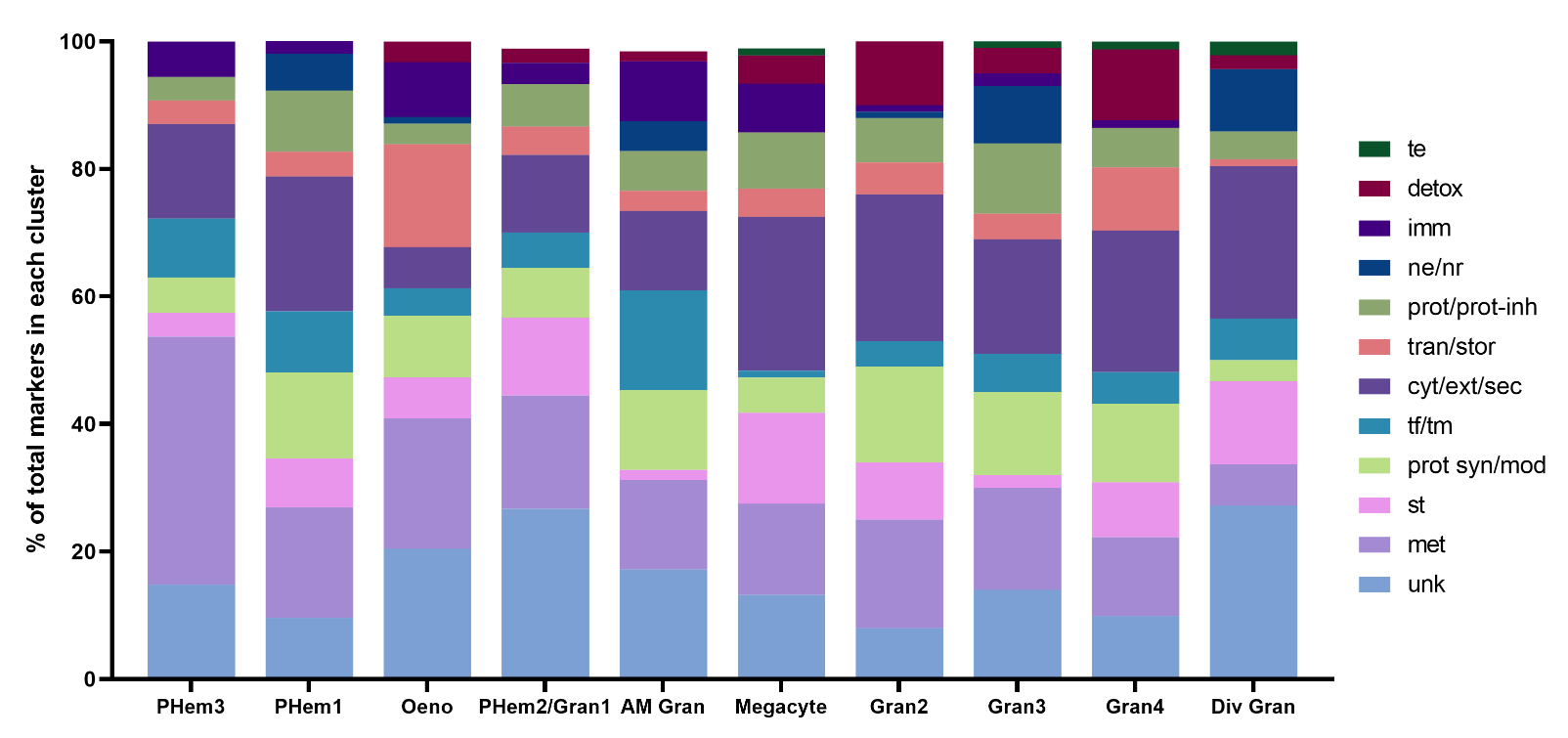


**Fig. S4** Functional differences between hemocyte subpopulations

Graph representing the relative abundance of the different functional classes of proteins encoded by the marker genes in each hemocyte cluster. (**unk**: unknown, **met**: metabolism, **st**: signal transduction, **prot syn/mod**: protein synthesis/modification, **tf/tm**: transcription factor/transcription machinery, **cyt/ext/sec**: cytoskeleton/extracellular matrix and adhesion/secreted, **tran/stor**: transport/storage, **ne/nr**: nuclear export/nuclear regulation, **prot/prot-inh**: protease/protease inhibitor, **imm**: immunity, **detox**: detoxification and oxidant metabolism, **te**: transposable element).

**Table S1.** **Read summary of the Illumina and PacBio libraries**

Table with the summary of length, % mapping, and quality of Illumina and PacBio reads from the libraries used to build the hemocyte transcriptome.

| **Sample** | **Total reads** | **Mapped reads** | **Mapped bases** | **Avg read length** | **Avg quality score (Phred)** | **% of mapped reads** |
| --- | --- | --- | --- | --- | --- | --- |
| **Illumina** |  |  |  |  |  |  |
| Cactus_Bound | 128,138,242 | 111,647,573 | 8,245,778,723 | 74 | 38.9 | 87.13 |
| Cactus_Unbound | 120,507,430 | 106,131,128 | 7,840,712,484 | 74 | 38.9 | 88.07 |
| HDF_Naive | 186,401,904 | 170,619,020 | 25,223,282,180 | 148 | 36.4 | 91.53 |
| HDF_Prime | 212,728,020 | 194,282,922 | 28,733,307,784 | 148 | 36.3 | 91.33 |
| LACZ_Bound | 151,872,372 | 130,956,908 | 9,672,347,920 | 74 | 38.9 | 86.23 |
| LACZ_Unbound | 53,246,656 | 47,219,745 | 3,465,889,616 | 73 | 38.4 | 88.68 |
| NP_Naive | 238,037,522 | 217,710,389 | 32,230,682,396 | 148 | 36.3 | 91.46 |
| NP_Prime | 243,985,982 | 222,592,462 | 32,970,996,736 | 148 | 36.3 | 91.23 |
| Sua_2dpi_Challenge | 229,481,778 | 206,405,606 | 20,555,855,047 | 99 | 36.2 | 89.94 |
| Sua_2dpi_Naive | 209,727,010 | 189,866,032 | 18,909,295,293 | 99 | 36.2 | 90.53 |
| Sua_4dpi_Challenge | 226,279,976 | 210,671,234 | 20,942,239,703 | 99 | 36.3 | 93.10 |
| Sua_4dpi_Naive | 256,131,486 | 236,295,938 | 23,511,575,414 | 99 | 36.2 | 92.26 |
| Sua_6dpi_Challenge | 229,982,042 | 205,113,019 | 20,466,720,753 | 100 | 36.2 | 89.19 |
| Sua_6dpi_Naive | 238,854,600 | 215,589,818 | 21,529,004,543 | 100 | 36.3 | 90.26 |
| **Total** | **2,725,375,020** | **2,465,101,794** | **274,297,688,592** | **105.93** | **36.99** | **90.07** |
| **PacBio** |  |  |  |  |  |  |
| HDF_Naive | 2,107,190 | 2,083,909 | 2,491,178,206 | 1,270 | 88.4 | 98.90 |
| HDF_Prime | 2,023,584 | 2,011,075 | 2,489,143,907 | 1,310 | 88.3 | 99.38 |
| Sua_2dpi_Challenge | 653,782 | 651,631 | 1,139,333,159 | 1,821 | 86.6 | 99.67 |
| Sua_2dpi_Naive | 627,788 | 620,702 | 1,226,013,318 | 2,053 | 87.3 | 98.87 |
| Sua_4dpi_Challenge | 1,316,778 | 1,299,739 | 2,413,272,801 | 1,926 | 86.3 | 98.71 |
| Sua_4dpi_Naive | 725,796 | 714,171 | 1,306,836,598 | 1,898 | 87.5 | 98.40 |
| Sua_6dpi_Challenge | 1,139,235 | 1,122,909 | 2,085,208,195 | 1,933 | 86.4 | 98.57 |
| Sua_6dpi_Naive | 916,822 | 901,617 | 1,648,209,385 | 1,896 | 87.5 | 98.34 |
| **Total** | **9,510,975** | **9,405,753** | **14,799,195,569** | **1,763** | **87** | **98.85** |

**Table S2. Predicted function of genes with multiple isoforms expressed in hemocytes.**

List of the functional categories of proteins which have multiple transcript variants expressed in hemocytes.

| **Predicted function of gene** | **No. of genes** |
| --- | --- |
| Putative serine/threonine-protein kinase | 66 |
| Putative Zinc finger | 22 |
| Hypothetical secreted protein | 20 |
| putative E3 ubiquitin ligase | 20 |
| Putative RNA recognition motif | 12 |
| Putative WD40 domain | 11 |
| putative btb | 8 |
| Putative family I63 unassigned peptidase inhibitors | 8 |
| Putative PDZ domain | 8 |
| hypothetical conserved secreted protein precursor | 7 |
| Putative ATP-binding cassette transporter family | 6 |
| Putative ankyrin repeats | 6 |
| Putative basic helix-loop-helix | 6 |
| Putative leucine-rich repeats | 6 |
| Putative Bromodomain | 5 |
| putative adenylate cyclase type 2 isoform | 4 |
| Putative Basic leucine zipper | 4 |
| Putative Broad-Complex | 4 |
| Putative Homeodomain | 4 |
| Putative Lysophospholipid Acyltransferases | 4 |
| Putative Major facilitator superfamily domain-containing14A and 14B | 4 |
| Putative Wings apart-like protein regulation of heterochromatin | 4 |
| Putative Formin Homology 2 Domain | 3 |
| Putative Histidine phosphatase superfamily | 3 |
| Putative K homology RNA-binding domain | 3 |
| Putative Kinesin motor domain | 3 |
| Putative Pleckstrin homology domain | 3 |
| Putative Septin | 3 |
| Putative SH3 domain-binding protein 5 | 3 |
| Putative SOX-TCF_HMG-box | 3 |
| Putative V ATP-ase 116kda subunit family | 3 |
| Other functional categories with two or less genes | 1162 |
| Hypothetical protein | 149 |
| Unknown product | 75 |

**Table S3. Function of immunity and transposable elements related proteins in hemocyte transcriptome.**

List of the predicted function of proteins in the hemocyte transcriptome related to immunity and transposable elements.

| **Predicted function** | **No. transcripts** |
| --- | --- |
| **Immunity** |  |
| Putative Immunoglobulin domain-containing protein | 52 |
| Putative ficolin | 49 |
| Putative lectin | 47 |
| Putative Peptidoglycan recognition protein | 33 |
| Putative SAM domain of SARM1-like | 30 |
| Putative Toll and Toll-interleukin 1 receptor domain-containing protein | 26 |
| Putative Integrin | 19 |
| Other functional categories | 229 |
| Total | 485 |
| **Transposable element** |  |
| Putative reverse transcriptase | 122 |
| Putative transposase | 43 |
| DDE endonuclease | 39 |
| Pol/Gag polyprotein | 37 |
| Putative transposable element | 21 |
| Putative retrotransposon peptidase | 17 |
| Other functional categories | 148 |
| Total | 427 |

**Annotation table of hemocyte transcripts**

The detailed annotation table can be downloaded as a single .ZIP file from the following link:

[https://proj-bip-prod-publicread.s3.amazonaws.com/transcriptome/An_gambiae_hemocytes_2022/AgHemocytes.zip](https://gcc02.safelinks.protection.outlook.com/?url=https%3A%2F%2Fproj-bip-prod-publicread.s3.amazonaws.com%2Ftranscriptome%2FAn_gambiae_hemocytes_2022%2FAgHemocytes.zip&data=05%7C01%7Cbanhisikha.saha%40nih.gov%7Ce397013b844346b2ca9308db9e7aff07%7C14b77578977342d58507251ca2dc2b06%7C0%7C0%7C638278023322426569%7CUnknown%7CTWFpbGZsb3d8eyJWIjoiMC4wLjAwMDAiLCJQIjoiV2luMzIiLCJBTiI6Ik1haWwiLCJXVCI6Mn0%3D%7C3000%7C%7C%7C&sdata=sAxIwD0CH8roQXF8fS%2BY2uTAU5eKbFMSiqW%2FcpB5pDo%3D&reserved=0).

CDS—Windows-compatible hyperlinked Excel file of the 31,375 CDS and their functional annotation.

lncRNA—Windows-compatible hyperlinked Excel file of 3203 lncRNAs with their length.

Short-ncRNA—Windows-compatible hyperlinked Excel file of 160 short ncRNAs with their length and functional category according to Rfam database.
